# Comparing Regularized Logistic Regression and Stochastic Gradient Descent in Predicting Drug-Gene Interactions of Inhibitors of Apoptosis Proteins in Periodontitis

**DOI:** 10.7759/cureus.70858

**Published:** 2024-10-04

**Authors:** Johnisha Harris, Pradeep Kumar Yadalam, Carlos M Ardila

**Affiliations:** 1 Periodontics, Saveetha Dental College and Hospital, Chennai, IND; 2 Periodontology, Saveetha Institute of Medical and Technical Sciences, Chennai, IND; 3 Basic Sciences, University of Antioquia, Medellin, COL

**Keywords:** apoptosis, drug-gene interactions, machine learning, periodontitis, predictive models

## Abstract

Objective: Periodontitis, characterized by inflammation linked to apoptosis dysregulation, underscores the role of inhibitors of apoptosis proteins (IAPs) like survivin and cIAP1, implicated in disease progression and treatment resistance across various conditions. Our study aims to analyze the prediction of drug-gene interactions by machine learning techniques, combining regularized logistic regression and stochastic gradient descent (SGD) for efficient classification.

Methods: Data from Probes-Drugs.org on IAP-based drug-protein interactions underwent rigorous annotation and outlier removal. A data robot tool trained machine learning models, regularized logistic regression and SGD (https://app.datarobot.com/new). Network analysis employed Cytoscape to construct and analyze the IAP network, identifying key hub nodes crucial in periodontitis pathogenesis.

Results: The constructed IAP network comprised 376 nodes and 556 edges, revealing intricate drug-gene interactions with an average of 2957 neighbors per node. Ten hub nodes were identified as pivotal in regulating biological processes specific to periodontitis, suggesting their potential as therapeutic targets and biomarkers. Predictive models demonstrated high accuracy, with gradient descent achieving 93% and regularized logistic regression achieving 92% in identifying drug-gene interactions within the IAP network.

Conclusions: These findings highlight the utility of computational methods in elucidating molecular mechanisms underlying periodontitis, offering insights into potential therapeutic strategies targeting IAP-related pathways. Future research should focus on validating hub genes experimentally and integrating multi-omics data to advance precision medicine approaches in periodontitis treatment.

## Introduction

Periodontitis is a condition characterized by inflammation that, if left untreated, can lead to tooth loss [1]. Symptoms include inflamed gums, halitosis, and gingival recession. While poor oral hygiene is the primary cause of periodontitis, certain factors such as smoking, diabetes, hormonal changes, and genetic predisposition can increase the risk. Apoptosis, also known as programmed cell death, plays a critical role in maintaining tissue homeostasis and regulating the inflammatory response in periodontitis, a chronic inflammatory disease affecting the supporting structures of the teeth [2].

Inhibitory apoptotic proteins (IAPs) are a group of cellular proteins that regulate apoptosis. These proteins control cell survival and prevent excessive or inappropriate cell death. IAPs primarily function by blocking the activity of caspases, which are enzymes essential for the execution of apoptosis. By inhibiting caspase activity, IAPs promote cell survival [3]. One of the most well-known IAPs is the cellular inhibitor of apoptosis protein 1 (cIAP1), which can bind to caspases and block their activation, thereby preventing apoptosis. Another important IAP is survivin, which is involved in cell division and also inhibits apoptosis. The IAP family in humans includes eight members, all containing a BIR domain, with XIAP being the most well-known for its role in inhibiting caspases and preventing apoptosis [4].

IAP proteins are crucial for maintaining cellular homeostasis and regulating apoptosis. Oral diseases such as periodontal disease and oral cancers often exhibit abnormal cell survival and resistance to apoptosis, which contributes to disease progression. The overexpression of IAP proteins, particularly those containing Baculoviral IAP repeat domains, has been linked to anti-apoptotic effects, posing significant challenges for therapeutic interventions [5].

In periodontitis, dysregulation of apoptosis is believed to contribute to disease progression. An imbalance between cell survival and death mechanisms may lead to the excessive destruction of periodontal tissues [6]. Several studies have investigated the role of IAPs in periodontitis, revealing that IAPs such as survivin and cIAP1 are upregulated in the periodontal tissues of patients with periodontitis. This upregulation might be a protective response to counteract the increased cell death in the inflamed periodontal tissues [7].

The integration of drug-gene interaction information into clinical decision support systems helps healthcare providers make personalized medication decisions [6]. Drug-gene interactions can occur through various mechanisms. Protein-coding interactions involve drugs interacting with specific genes to affect protein synthesis. Gene fusions create chimeric proteins, which can be targets for drugs [8]. Protein-protein interactions involve drugs targeting specific interactions between proteins, while single-protein interactions involve drugs targeting individual proteins. Additionally, drugs can target entire protein families, affecting multiple proteins with similar functions or structures [9].

Machine learning techniques can predict drug-gene interactions by analyzing large datasets of genomic and drug response information [10]. Machine learning algorithms can identify patterns and relationships to predict new drug-gene interactions by training models on known interactions [11]. Regularized logistic regression is a classification algorithm that adds a regularization term to the cost function to prevent overfitting [12]. Stochastic gradient descent (SGD) is an optimization algorithm that updates the model parameters based on the gradients of randomly selected training examples. Combining regularized logistic regression with SGD allows for efficient and accurate classification while avoiding overfitting. Predicting drug-gene interactions is crucial for drug selection, as it helps avoid toxicity and enhances potency.

Despite advancements in the use of machine learning for drug-gene interaction prediction, existing models often suffer from overfitting or inefficiency when dealing with large datasets, especially in diseases with complex pathophysiology like periodontitis. This study fills the gap by comparing two powerful models, regularized logistic regression and SGD, to enhance prediction accuracy and efficiency, thus providing a more robust approach to drug-gene interaction analysis in periodontitis. This model aims to improve current practices by mitigating the limitations of overfitting and optimizing computational resources.

This study aims to compare regularized logistic regression and SGD in predicting drug-gene interactions of IAPs in periodontitis.

## Materials and methods

Using data from Probes & Drugs (https://www.probes-drugs.org/), drug-gene interactions were downloaded [13]. The 557, drug dataset was annotated, pre-processed, and outliers were removed for protein interactions. This annotated dataset of inhibitors of the apoptosis family of proteins contains information such as ID name, biochemical activity, mode of action, and drug-gene interaction types, including protein-coding, chimeric protein, protein-protein interactions, single protein, and protein family [14]. Using Cytoscape, a drug-gene interaction network was constructed for drugs and genes with biochemical interaction types, and hub genes were identified for network analysis [15].

With the DataRobot tool, protein interactions were used as targets to analyze the accuracy of regularized logistic regression and SGD. A confusion matrix was created to evaluate the performance of these methods.

Regularized logistic regression is a variant of logistic regression that incorporates a regularization term into the cost function [16,17]. The purpose of regularization is to prevent overfitting and enhance the model's generalization ability. In regularized logistic regression, the cost function is adjusted to include a regularization term that penalizes large weight values. This adjustment helps control the model's complexity and prevents it from fitting the noise in the training data.

The regularization term is added to the cost function as the last term to penalize large weight values. The regularization parameter controls the strength of the regularization and must be carefully chosen to balance fitting the training data and avoiding overfitting. During training, the weights are updated using gradient descent or another optimization algorithm that minimizes the cost function. The gradient of the cost function with respect to the weights is computed, and the weights are updated in the opposite direction of the gradient.

The architecture of regularized logistic regression consists of several key components, each contributing to the model's functionality. These components work together to process input data, apply a regularization mechanism to prevent overfitting, and iteratively adjust model parameters using gradient-based optimization methods. Table 1 below outlines these core elements in detail.

**Table 1:** Key components of the regularized logistic regression architecture SGD, stochastic gradient descent

Regularized Logistic Regression Architecture
Input Layer: Represents the features or inputs for each training example, typically as a vector or matrix.
Weight Vector: Contains the coefficients or parameters learned during training.
Bias Term: Additional parameter that accounts for any offset in predictions, added to the weighted sum of inputs.
Activation Function: Sigmoid function, mapping the weighted sum of inputs to a value between 0 and 1, representing class probability.
Regularization Term: Penalizes large weights in the cost function to prevent overfitting and control model complexity.
Cost Function: Consists of the negative log-likelihood loss and regularization term, measuring the error between predicted and actual labels.
Gradient Descent Optimization: Uses SGD to update weights and bias iteratively based on cost function gradients.

SGD is an optimization algorithm commonly used in machine learning, especially for large-scale datasets [17]. It is a variant of gradient descent that updates the model parameters (weights) on each training example rather than using the entire batch of training examples simultaneously. The main idea behind SGD is to estimate the gradient of the cost function using a randomly selected subset of the training data, also known as a mini-batch. This makes the computation more efficient, requiring less memory and computational power than batch gradient descent. Additionally, by randomly selecting the training examples in each iteration, SGD introduces some "noise" into the optimization process, which can help the algorithm escape local minima and achieve better exploration of the parameter space.

SGD continues to iterate over the training examples until convergence or a maximum number of iterations is reached. At each iteration, it randomly selects a mini-batch of training examples and updates the weights based on the gradient computed from it. SGD is computationally efficient and particularly useful when dealing with large datasets, as it processes the data in small chunks rather than all at once. However, it can be sensitive to the learning rate and may require careful tuning to ensure convergence. Additionally, as the updates are based on a single example or a mini-batch, the optimization process may exhibit more variability and noise than batch gradient descent.

SGD is a widely used optimization algorithm in machine learning models, including neural networks. It operates by updating weights iteratively based on gradients computed from small batches of training data. Table 2 provides a breakdown of the essential elements of the SGD architecture, highlighting the steps involved in gradient computation, weight updates, and convergence monitoring. 

**Table 2:** Key components of the SGD architecture SGD, stochastic gradient descent

SGD Architecture
Input Layer: Initial layer where input data is fed into the network.
Hidden Layers: Intermediate layers where data is transformed through activation functions and weights.
Output Layer: Final layer where the network makes predictions.
Cost Function: Objective function measuring the difference between predicted and true outputs, guiding weight updates.
Gradient Computation: Gradient is computed for each training example or mini-batch, propagated backward through the network.
Weight Update: Weights are updated using the gradient and learning rate to minimize the cost function.
Mini-Batch Selection: A random mini-batch of training examples is selected for gradient computation and weight updates.
Convergence: SGD iterates until convergence or a maximum number of iterations, monitored by changes in the cost function or weights.

Cytoscape

Cytoscape (Cytoscape Consortium, 2016) is a popular open-source software platform for visualizing and analyzing biological networks such as protein-protein interaction networks, gene regulatory networks, and signaling networks [15,18,19]. It provides a user-friendly interface and a wide range of tools and plugins for network analysis and visualization. Cytoscape includes a plugin called cytoHubba, specifically designed for network analysis tasks such as identifying important or hub nodes. cytoHubba provides various algorithms and methods for network topology analysis and node ranking.

## Results

**Figure 1:**
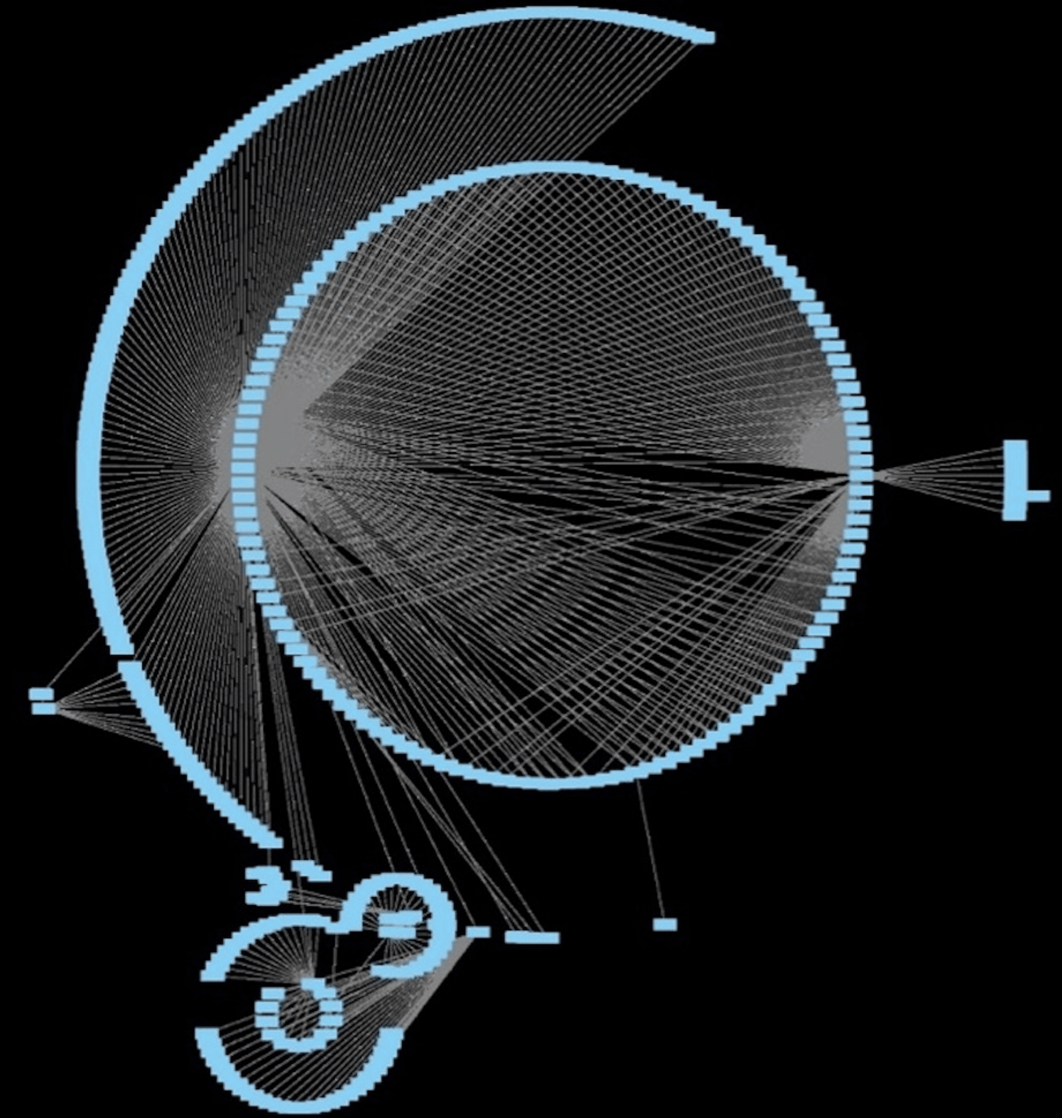
IAPs IAPs, inhibitors of apoptosis proteins

Network analysis of drug-gene interactions with protein interactions revealed a network consisting of 376 nodes and 556 edges, with an average number of neighbors being 2957. Ten hub nodes were identified using the cytoHubba plugin (Figure 1).

Figure 1 depicts IAPs, illustrating a densely interconnected network of drug-gene interactions within the protein family. The figure serves to elucidate the molecular architecture and functional mechanisms of IAPs, providing insights into their potential therapeutic relevance in apoptosis-related diseases.

Hub genes are pivotal elements within biological networks or systems, characterized by their extensive connectivity and involvement in numerous network interactions. These genes, commonly referred to as "hubs," play crucial roles in regulating biological processes and diseases. The concept of hub genes is widely employed in network and systems biology research to identify key drivers or regulators of these processes. Researchers utilize various methods and algorithms to pinpoint hub genes, typically examining network topology, centrality measures, and gene expression data. Common metrics for identifying hub genes include degree centrality, betweenness centrality, closeness centrality, eigenvector centrality, and module membership. Once identified, hub genes undergo detailed study to elucidate their biological functions, regulatory mechanisms, and potential implications in disease pathology. They often serve as promising targets for therapeutic intervention or biomarkers across a spectrum of diseases, such as cancer, neurological disorders, and immune-related conditions (Table 3). Table 3 presents the primary hub drugs and genes within the inhibitors of the apoptosis proteins family.

**Table 3:** Top hub drugs and genes in the IAPs family IAPs, inhibitors of apoptosis proteins

Serial	Gene Name	Role in Apoptosis
1	Birinapant	Inhibitor of apoptosis
2	Estrogen receptor	Involved in apoptosis regulation
3	Baculoviral inhibitor of apoptosis repeat-containing protein 7	Inhibitor of apoptosis
4	Baculoviral inhibitor of apoptosis repeat-containing protein 5	Inhibitor of apoptosis
5	Baculoviral inhibitor of apoptosis repeat-containing protein 3	Inhibitor of apoptosis
6	E3 ubiquitin-protein ligase XInhibitor of apoptosis	Inhibitor of apoptosis
7	Baculoviral Inhibitor of apoptosis repeat-containing protein 2	Inhibitor of apoptosis
8	Dequalinium	Induces apoptosis in cancer cells
9	Embelin	Induces apoptosis through XInhibitor of apoptosis inhibition
10	Phenylalanine	Precursor for protein synthesis

Confusion matrix

A confusion matrix is a table that evaluates the performance of a classification model [20,21]. It summarizes the predictions made by the model on a test dataset by comparing them to the actual labels. 1. True positive (TP): The model correctly predicted the positive class. 2. True negative (TN): The model correctly predicted the negative class. 3. False positive (FP): The model incorrectly predicted the positive class when the actual label was negative (type I error). 4. False negative (FN): The model incorrectly predicted the negative class when the actual label was positive (type II error).

Lift chart

A lift chart, also referred to as a gains chart or cumulative gains chart, is a graphical representation used to evaluate the performance of a predictive model, especially in applications like customer response modeling or churn prediction [22,23].

In a lift chart, the x-axis typically represents the cumulative percentage of the population, ordered by the model's predicted probability or score. The y-axis shows the cumulative percentage of positive instances captured (such as responses or events of interest). The lift or gain is calculated as the ratio between the cumulative percentage of positive instances captured by the model and the expected percentage if no models were used.

Plotting the lift curve allows us to assess the model's effectiveness compared to a baseline scenario (often random or no model). A higher lift value at the top percentages of the population indicates that the model captures a greater proportion of positive instances than the baseline. This comparison helps determine whether the model adds value in identifying positive instances more effectively than random guessing or no model at all.

**Figure 2:**
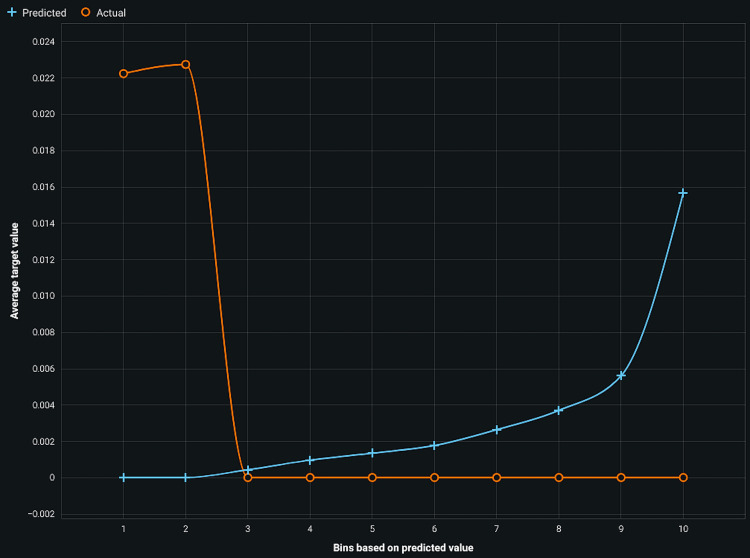
Lift chart of all classes in logistic regression

Figure 2 displays the lift chart for all classes in logistic regression with a high lift value. The lift chart helps assess how well the model identifies positive instances compared to a random selection, providing insights into its effectiveness for different classes within the dataset.

**Figure 3:**
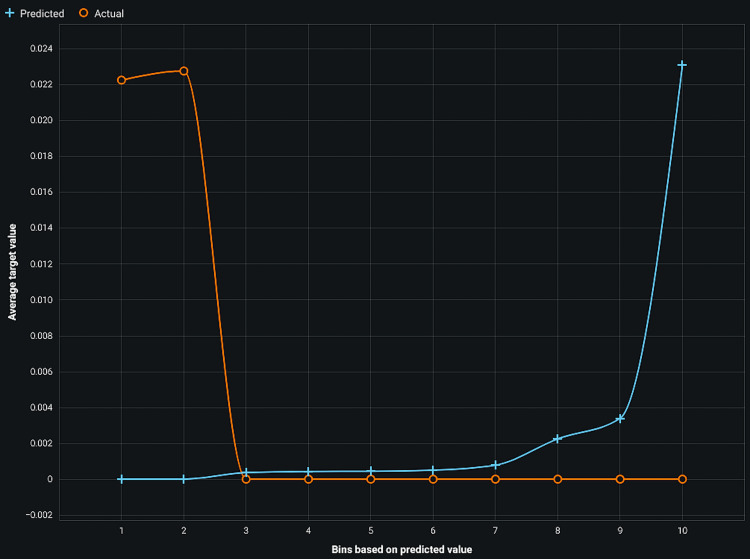
Lift chart of all classes of gradient descent

Figure 3 presents the lift chart for all classes in gradient descent with a high lift value.

The lift chart provides a clear visualization of how well the gradient descent model identifies positive instances compared to random selection, highlighting its efficacy across different classes within the dataset. The accuracy of gradient descent is 93%, and for regularized logistic regression, it is 92%.

## Discussion

IAPs are proteins containing the baculovirus IAP repeat (BIR) domain, which facilitates zinc-binding and protein-protein interactions [24]. In humans, there are eight distinct IAPs, each typically harboring one to three BIR domains [25]. Certain IAPs, such as DIAP2 and XIAP, also feature a Ub-associated domain (UBA) for poly-Ub chain binding and a RING domain for E3 ligase activity. cIAP1 and cIAP2 additionally possess a caspase recruitment domain (CARD) that can modulate their E3 ligase function. Notably, SOD2 and BIRC3 have been identified as inhibitors of apoptosis [26]. Previous studies have reported elevated gene expression and protein levels of SOD2 and BIRC3 in patients with periodontitis compared to healthy individuals, suggesting their pivotal role in periodontal infection and inflammation. The upregulation of SOD2 and BIRC3 may serve as a protective mechanism against the loss of periodontal cells and tissues [5,9].

Members of the Bcl-2 family, such as Bcl-2, Bcl-xL, and Mcl-1, can have their anti-apoptotic functions inhibited by small compounds or peptides. IAP proteins like survivin, cIAP, and XIAP regulate caspase activity to prevent apoptosis. Caspases are critical mediators of apoptosis [27]. For instance, Z-VAD-FMK, a small molecule caspase inhibitor, blocks apoptosis by inhibiting catalytic caspase activity. The protein FLIP also prevents apoptosis by inhibiting caspase-8, a key initiator of caspase.

In this study, a significant hub gene identified is Birc7, also known as Livin, a member of the IAP family [25]. Livin plays a crucial role in regulating apoptosis, cell proliferation, and immune responses. Understanding its functions provides insights into various cellular processes.

Recent research has advanced our understanding of complex interactions within cell death signaling pathways. Caspase-8, a pivotal regulator of apoptosis, necroptosis, and pyroptosis, is activated in response to LPS binding to TLRs, triggering necroptosis involving RIPK1 and RIPK3. IAP proteins in mammals can inhibit apoptotic caspases, potentially leading to increased necroptosis and activation of NLRP3/IL-1β. In periodontitis, survivin and XIAP prolong the lifespan of inflammatory cells [26]. Dysregulation of caspase-8 enhances necroptosis and pyroptosis, while c-FLIP activation suppresses these pathways. The intricate interplay between cell death mechanisms suggests differential susceptibility of various periodontal cell types during periodontitis.

This study employs gradient descent and regularized logistic regression to explore protein coding, chimeric protein interactions, and drug-gene interactions [28]. Predicting these interactions is critical for developing drugs with enhanced efficacy and fewer side effects. Prior research has utilized deep learning methods like PINNACLE and ChiPPI to generate context-aware protein representations and identify therapeutic targets [29]. Recent advances in deep learning architectures based on graph representation learning have demonstrated superior performance in predicting protein interaction sites and interactions across diverse datasets [30].

The study reports an accuracy of 93% for gradient descent and 92% for regularized logistic regression, supported by lift charts indicating robust model performance (Figures 2, 3). However, future research should focus on larger datasets and improved algorithms to enhance predictive capabilities and facilitate clinical applications in treating periodontal diseases by targeting apoptosis inhibition.

Limitations of the study

Although this study contributes to the understanding of protein interactions and drug-gene associations in periodontitis, several limitations should be acknowledged. First, the dataset used in this study was relatively small, which may limit the generalizability of the results. Second, the study focused primarily on gradient descent and regularized logistic regression, and exploring other machine-learning algorithms may provide additional insights. Third, the study did not validate the predicted interactions experimentally, which is essential for confirming the accuracy of the predictions.

## Conclusions

The predictions stemming from drug-gene interactions involving inhibitors of apoptosis offer potential insights into therapeutic targets and the pharmacological mechanisms driving apoptosis in periodontal disease. These findings may help in identifying novel drug candidates or repurposing existing therapies to target key apoptotic pathways, potentially leading to more effective treatments for periodontitis. Moving forward, it is crucial to validate these predictions through rigorous preclinical and clinical studies to substantiate their clinical relevance. In particular, future studies should focus on evaluating the safety and efficacy of these potential therapeutic targets in human subjects, which could pave the way for personalized treatment approaches and improve patient outcomes in periodontal disease management.
